# A New Self-Expandable Metallic Stent with Low Axial Force and a High Axial Force Zero-Border Shows a Very Low Perforation Rate in Malignant Colorectal Obstruction: A Japanese Multicenter Prospective Study

**DOI:** 10.3390/jcm13175102

**Published:** 2024-08-28

**Authors:** Takashi Murakami, Hiroyuki Isayama, Satoshi Ikeda, Norihiro Hanabata, Toshiyuki Enomoto, Toshio Kuwai, Mitsunori Ushigome, Masahide Ebi, Hideo Ohtsuka, Shungo Endo, Shuji Saito, Takeshi Ohki, Ryuichi Yamamoto, Takahisa Kayahara, Satoshi Matsumoto, Yoshihiro Sasaki, Yoshihisa Saida

**Affiliations:** 1 Department of Gastroenterology, Graduate School of Medicine, Juntendo University, Tokyo 113-8421, Japan; t-murakm@juntendo.ac.jp; 2 Department of Gastroenterological Surgery, Hiroshima Prefectural Hospital, Hiroshima 734-8530, Japan; sikeda1965@way.ocn.ne.jp; 3 Department of Gastroenterology, Aomori Prefectural Central Hospital, Aomori 030-0913, Japan; ohana@pastel.ocn.ne.jp; 4 Department of Surgery, Toho University Ohashi Medical Center, Tokyo 153-8515, Japan; eno@med.toho-u.ac.jp (T.E.); yoshisaida@nifty.com (Y.S.); 5 Gastrointestinal Endoscopy and Medicine, Hiroshima University Hospital, Hiroshima 734-0037, Japan; toshiokuwai@gmail.com; 6 Department of Gastroenterology, NHO Kure Medical Center and Chugoku Cancer Center, Hiroshima 737-0023, Japan; 7 Department of General and Gastroenterological Surgery, Toho University Omori Medical Center, Tokyo 143-8541, Japan; ushisan@med.toho-u.ac.jp; 8 Department of Gastroenterology, Aichi Medical University School of Medicine, Nagakute 480-1155, Japan; mebi@aichi-med-u.ac.jp; 9 Department of Surgery, Tokyo Metropolitan Tama Medical Center, Tokyo 189-8511, Japan; hideo_ootsuka@tmhp.jp; 10 Department of Coloproctology, Aizu Medical Center, Fukushima Medical University, Fukushima 969-3482, Japan; endoswing@gmail.com; 11 Division of Surgery, Gastrointestinal Center, Yokohama Shin-Midori General Hospital, Kanagawa 226-0025, Japan; shusaito@jb3.so-net.ne.jp; 12 Department of Surgery, Institute of Gastroenterology, Tokyo Women’s Medical University, Tokyo 162-8666, Japan; ohki.takeshi@twmu.ac.jp; 13 Department of Gastroenterology, Tokyo-West Tokushukai Hospital, Tokyo 196-0003, Japan; ryuichi5118@gmail.com; 14 Department of Gastroenterology and Hepatology, Kurashiki Central Hospital, Okayama 710-0052, Japan; tk13831@kchnet.or.jp; 15 Department of Gastroenterological Surgery, Nippon Medical School Chibahokusoh Hospital, Chiba 270-1694, Japan; s-matsu@nms.ac.jp; 16 Department of Gastroenterology, National Hospital Organization Disaster Medical Center, Tokyo 190-0014, Japan; ezl06015@nifty.ne.jp

**Keywords:** malignant colorectal obstruction, self-expandable metallic stent, JENTLLY, axial force, axial force zero-border

## Abstract

**Background:** Recently, there has been a significant increase in the utilization of self-expandable metallic stents (SEMSs) for treating malignant colorectal obstructions through colorectal stenting. The mechanical properties of SEMSs are usually considered to affect clinical outcomes of patients with malignant colorectal obstructions. **Methods**: This single-arm, prospective, multicenter study of SEMS with a lower axial force and high axial force zero-border included 200 patients with malignant colorectal obstruction. Technical and clinical success, stent patency, and adverse events associated with SEMS placement were evaluated. **Results:** One patient was excluded, and 199 patients were evaluated. The treatment intent was bridge-to-surgery in 129 and palliation in 70 patients. Technical and clinical success rates were 99.5% and 97.0%, respectively. The percentage of the ColoRectal Obstruction Scoring System scores of 3 or higher improved significantly from 19.2% before placement to 93.9% after placement. Clinical success was not achieved in five patients due to insufficient stent expansion in four patients and stent occlusion in one patient. Only one patient underwent emergency surgery for perforation of the proximal colon, far from where the stent was placed; the rescue procedure was not performed, despite no improvement in proximal dilatation due to insufficient stent expansion. Among the palliation cohort, 15 patients received chemotherapy, including molecular-targeted agents such as bevacizumab. There were no fatal cases related to stent placement. **Conclusions**: For management of malignant colorectal obstruction, this newly developed SEMS with low axial force and a high axial force zero-border showed high technical and clinical success rates, and an extremely low perforation rate (0.5%).

## 1. Introduction

Colorectal cancer is prevalent globally, particularly in economically developed nations. In 8–13% of colorectal cancer patients, the bowel becomes obstructed [1]. The two main sources of malignant obstruction are primary colorectal cancer and extra-colonic malignancies such as gastric, gynecologic, and pancreaticobiliary tumors. The symptoms include abdominal pain, bowel dilatation, nausea, and vomiting. Urgent decompression is necessary to prevent bacterial translocation, colonic necrosis, electrolyte and fluid imbalances, and perforation.

Endoscopic colorectal stenting with self-expandable metallic stents (SEMSs) is commonly used to provide symptomatic relief [2]. Colorectal stent placement is widely embraced for bridge-to-surgery (BTS) and palliative care (PAL). SEMS insertion for BTS can prevent the need for a stoma and allow for preoperative oral intake. Colorectal stenting as a minimally invasive intervention for PAL also preserves quality of life. Colorectal stenting is a minimally invasive, valuable option for the treatment of malignant colorectal obstructions. Although stenting offers benefits, such as avoiding emergency surgery and providing immediate symptom relief, several challenges and areas for improvement remain unresolved. Despite advancements, colorectal stent placement has complications, including stent occlusion, stent migration, colorectal perforation, and stent-related symptoms such as pain and tenesmus. Addressing these complications effectively requires the ongoing refinement of optimal patient selection, deployment techniques, postprocedural management, and SEMS design.

To reap these benefits, safe and effective stent placement leading to sustained luminal patency is crucial. Affecting long- and short-term outcomes, bowel perforation is a serious life-threatening complication of colorectal stent placement [3]. One reason for perforation following introduction of the SEMS is the constant mucosal contact with the SEMS. Additionally, the SEMS exerts continuous radial and axial pressure on the colonic wall. The mechanical properties of SEMSs, including these radial and axial forces, are thought to impact the clinical outcomes of biliary and esophageal stenting [4,5]. Radial force is further divided into the radial resistance force (which maintains the stent’s shape against external pressure) and the radial expansion force (which is generated by stent expansion from a collapsed state) [6]. SEMSs with lower radial force might not sufficiently dilate the stricture, whereas those with higher radial force might cause perforation due to excessive pressure on the colonic wall. On the other hand, axial force is created by the straightening of a bent SEMS. A high axial force is thought to be a determinant of gastrointestinal tract perforation [7]; thus, lower axial force is expected to reduce the risk of perforation. In a recent study, we introduced a novel mechanical property called the axial force zero-border, defined as the angle at which the axial force disappears [8]. SEMSs with a high axial force zero-border do not continuously compress the gastrointestinal wall, and are considered to be at low risk for causing perforation. SEMSs with hook- and cross-type structures have a relatively high axial force zero-border [8].

**Figure 1:**
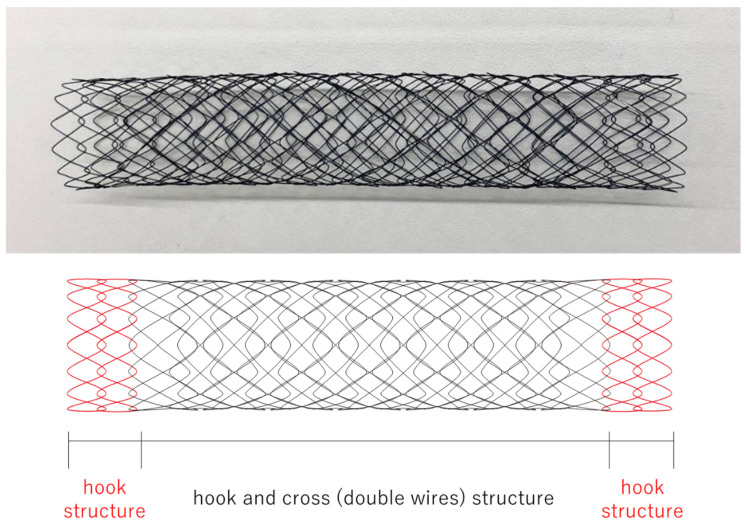
JENTLLY Colonic Stent (Japan Lifeline Co., Ltd., Tokyo, Japan). The JENTLLY Colonic Stent is a self-expandable uncovered nickel-titanium alloy stent (SEMS). Its hook- and cross-type design results in a relatively high axial force zero-border and lower axial force.

No consensus has been reached on which type of SEMS is the best. The JENTLLY Colonic Stent (Japan Lifeline Co., Ltd., Tokyo, Japan) is an uncovered SEMS made from nitinol (a metal alloy of nickel and titanium) (Figure 1). Its hook- and cross-type design results in a relatively high axial force zero-border and lower axial force [8]. We conducted a multicenter, prospective study of the placement of this colonic SEMS. This study focused on evaluating the short-term efficacy and safety of these SEMSs for malignant colorectal obstruction management.

## 2. Materials and Methods

### 2.1. Study Design

In this multicenter (15 academic medical centers and community hospitals) study by the Japan Colonic Stent Safe Procedure Research Group (JCSSPRG), we prospectively examined the safety and effectiveness of colorectal stenting with an uncovered SEMS, specifically the JENTLLY Colonic Stent. Prior to enrollment, all patients gave their informed consent to participate, and the institutional review board at each participating institution gave its approval. The study was registered in the University Hospital Medical Information Network Clinical Trial Registry (UMIN000032044).

Patients eligible for inclusion had malignant large bowel obstruction requiring colonic stent placement, confirmed by imaging studies such as computed tomography, colonoscopy, or abdominal radiography. Patients were excluded for the following reasons: endoscopic treatment contraindicated, enteral ischemia, intra-abdominal abscess, past history of colonic stenting, severe inflammatory changes around the obstruction, and suspected or impending perforation. These criteria helped ensure patient safety and procedural success, as these patients could have been at risk of further complications, such as perforation, either during the procedure or after stent placement. Each patient was registered prior to SEMS placement. The clinical data were prospectively gathered. SEMSs were installed as BTS in patients scheduled for surgery, and as PAL treatment in those not scheduled for surgery. The study evaluated the short-term safety and efficacy of colonic stenting using the JENTLLY Colonic Stent, with a follow-up period of up to five years from the stent placement date.

### 2.2. Stent Placement

The stents were placed under fluoroscopic guidance, through the colonoscope channel. Before this study began, group members were given tips for stent placement, and points to avoid complications. Following “the JCSSPRG Mini-Guidelines” [9], the colonoscope was gently inserted and advanced up to the colorectal obstruction site; the guidewire and catheter were maneuvered through the narrowed passage; contrast medium was injected through the catheter to highlight the stricture; the obstruction length was fluoroscopically measured; and the SEMS was deployed into the stricture under fluoroscopic and endoscopic guidance using the through-the-scope method. The guidelines recommended using clips for intraluminal marking of the stricture location, and avoiding stricture dilatation to prevent perforation. SEMS placement was performed or supervised by experienced endoscopy experts from the JCSSPRG. In this study, the evaluation was limited to uncovered colonic stents, specifically the JENTLLY Colonic Stent. These stents are 18 or 22 mm in diameter, 6, 8, 10, or 12 cm in length, and packaged in 9 or 10 Fr. delivery systems.

### 2.3. Clinical Outcomes

The ColoRectal Obstruction Scoring System (CROSS) was created and defined by the JCSSPRG, and drew inspiration from a system designed for scoring malignant gastric outlet obstruction [2,10]. Oral intake, stricture symptoms, and the need for continuous decompression were scored as follows: CROSS 0, requiring continuous decompression; CROSS 1, no oral intake; CROSS 2, liquid or enteral nutrient intake; CROSS 3, soft solids, low residue, and full diet with stricture symptoms; or CROSS 4, soft solids, low residue, and full diet without stricture symptoms. The successful deployment of the stent across the entire length of the stricture was considered to be a technical success. The resolution of symptoms and radiological relief of the obstruction within 24 h, confirmed by radiographic observation, was considered to be a clinical success. After SEMS placement, complications including abdominal pain, bleeding, fecal incontinence, infection or fever, perforation, recurrent colorectal obstruction, stent migration, tenesmus, and other minor complications were evaluated. We defined bleeding as the requirement for a therapeutic intervention, early onset as onset within 7 days, and late onset as onset after 7 days. To calculate the incidence rate, we used the number of patients enrolled in all cohorts as the denominator.

### 2.4. Data Collection

An electronic registration system was used for prospective recording of all the clinical data. At enrollment, the patient’s age, CROSS score, Eastern Cooperative Oncology Group (ECOG) performance status (PS), etiology, sex, and symptoms of obstruction were collected and evaluated. Tumor characteristics included the number of obstructions, location of the obstruction stricture length, and type of obstruction. Outcomes assessed included causes of clinical failure, stent diameter and stent length, the number of stent placements, procedure time, and technical and clinical success. Safety was evaluated by investigating complications and the impact of concurrent chemotherapy.

### 2.5. Statistical Analysis

Categorical data are presented as absolute numbers and percentages; continuous data are presented as medians and ranges. The risk factors for (determinants of) perforation were analyzed using the chi-square test and model of logistic regression analysis, which included variables such as age, chemotherapy following SEMS placement, etiology, sex, stent length, and type of obstruction. All statistical analyses were conducted using JMP Pro software (version 16.0; SAS Institute, Chicago, IL, USA).

## 3. Results

### 3.1. Characteristics of the Patients

**Figure 2:**
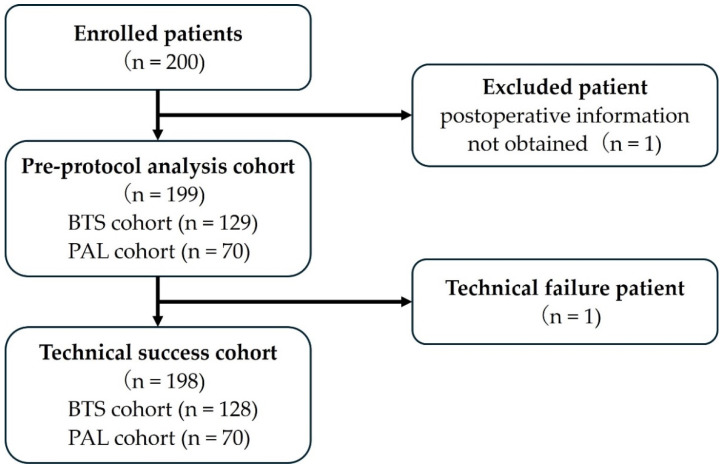
A flow chart of enrolled patients.

Figure 2 shows a flowchart of the patient registration process.

Two-hundred patients were enrolled between March 2018 and October 2022. One patient who underwent surgery at another hospital after stenting and for whom detailed postoperative information could not be obtained was excluded. Finally, this study included 199 patients. Table 1 shows the characteristics of the patients in this cohort.

The median age was 73.4 years, and 102 (55.5%) patients were male. The ECOG-PS score was 0 or 1 in 75% of the patients and 2 or higher in the other 25%. Eighty-three percent of patients had American Society of Anesthesiologists (ASA)-PS scores of 1 or 2, and the remaining seventeen percent had ASA-PS scores of 3 or 4. One-hundred and ninety-two patients (96.5%) had abdominal symptoms, including abnormal bowel movement (90.5%), bloating (79.4%), abdominal pain (67.8%), nausea (43.7%), and vomiting (27.1%). The most common etiology was colorectal cancer (*n* = 189; 95.0%), followed by gastric cancer (*n* = 4; 2.0%), pancreatic cancer (*n* = 4; 2.0%), bile duct cancer (*n* = 1; 0.5%), and bladder cancer (*n* = 1; 0.5%).

The treatment intent was BTS and PAL in 129 (64.8%) and 70 (35.2%) patients, respectively. The proportions of obstructions extending from the cecum to the transverse colon (right-sided), extending from the splenic flexure to the sigmoid colon (left-sided), and in the rectum were 26.6%, 51.8%, and 21.6%, respectively. Complete obstruction was observed in 175 (87.9%) patients. The digestive tract was decompressed before stent placement in 25 (12.5%) patients using a nasogastric tube (5.0%), transnasal ileus tube (4.0%), or transanal ileus tube (3.5%).

### 3.2. Clinical Outcomes of Stenting in the Cohort

Stenting in 198 (99.5%) patients was technically successful and had a mean procedure time of 30.0 min (Table 2). Technical failure of stenting occurred when the stent could not be delivered through the scope. No balloon dilatation of the stricture was performed prior to stent placement. During stenting, the mean stricture length was 4.3 cm. One-hundred and eighty-nine (95.0%) patients required only one SEMS, whereas ten (5.0%) patients required two SEMSs. In the first stent cohort, most commonly, we used stents that were 8 cm in length (42.2%) and 22 mm in diameter (72.9%).

**Figure 3:**
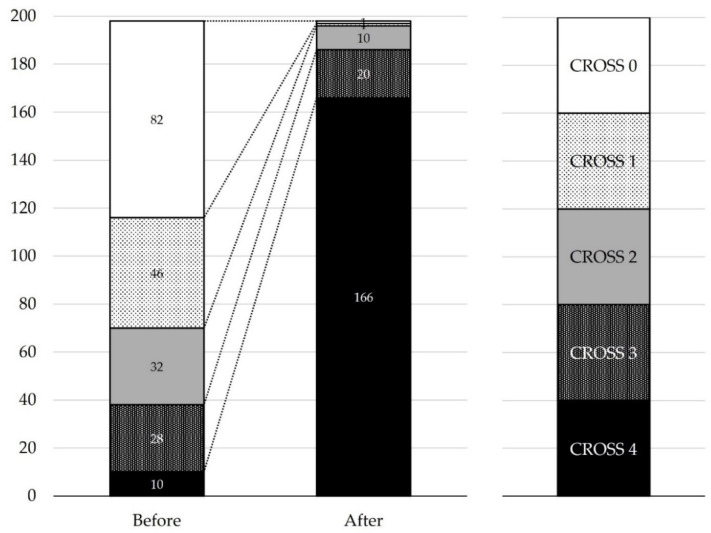
Comparison of ColoRectal Obstruction Scoring System (CROSS) scores before and after stent placement (n = 198). The CROSS scores were 0, 1, 2, 3, and 4 in 82 (41.4%), 46 (23.2%), 32 (16.2%), 28 (14.1%), and 10 (5.1%) patients, respectively, before stent placement, and in 1 (0.5%), 1 (0.5%), 10 (5.1%), 20 (10.1%), and 166 (83.8%) patients, respectively, after stent placement.

Treatment succeeded clinically in 193 (97.0%) patients. There were no fatalities due to colonoscope insertion or catheter or guidewire manipulation in this series. Excluding the one case of technical failure, the procedure failed clinically in five patients because of insufficient stent expansion (n = 4) or stent occlusion (n = 1). In two of the insufficient stent expansion cases, an additional SEMS was added. A transanal ileus tube was inserted in another patient before surgery. In the remaining case, there was an impending perforation due to obstruction by colonic cancer in the splenic flexure, with marked dilatation of the small intestine and proximal colon. The stent was successfully placed; however, the dilatation did not improve. Radiography showed insufficient stent expansion; however, an additional rescue procedure was not performed because of slight improvement in the patient’s symptoms. Two days after stent placement, perforation was confirmed in the cecum far from where the stent was placed, leading to surgery. An endoscopy without further intervention was used to manage other cases of fecal impaction. The CROSS scores before stent placement were 0, 1, 2, 3, and 4 in 82 (41.4%), 46 (23.2%), 32 (16.2%), 28 (14.1%), and 10 (5.1%) patients, respectively, and after stent placement they were 3 or higher in 186 (93.9%) patients (Figure 3).

### 3.3. Clinical Outcomes in the BTS Cohort

Treatment succeeded in 126 (98.4%) patients in the BTS cohort (n = 128). All enrolled patients were assessed for adverse events (Table 3). Ten (7.8%) early adverse events were observed, including abdominal pain in four, stent occlusion in four, fever in two, obstructive colitis in one, perforation in one, and stent migration in one patient. As mentioned above, a perforation developed in one patient 2 days after stent placement. The perforation was unrelated to stent placement, and the patient underwent emergency surgery. One patient with stent occlusion required emergency surgery. An additional SEMS was added in one case of stent occlusion. A transanal ileus tube was inserted before surgery in one patient with stent occlusion. In one patient, fecal impaction-induced stent occlusion was endoscopically managed without further intervention. All remaining patients with early adverse events were followed-up conservatively.

Between 8 and 20 days after stent placement, twelve (9.4%) late adverse events became apparent, including stent occlusion in five, fever in three, stent migration in three, abdominal pain in one, and obstructive colitis in one. Transanal and transnasal ileus tube insertions were performed in each case with stent occlusion before surgery. The remaining three patients with stent occlusion were followed-up conservatively until surgery. All patients with stent migration, abdominal pain, and obstructive colitis were followed-up conservatively until surgery.

The mean interval from stent placement to surgery was 27.6 days, and the number of patients scheduled to receive elective surgery was 126 (98.4%), which did not include one patient with perforation and one patient with stent occlusion, as described above. In the BTS cohort, 103 (80.5%) patients underwent laparoscopic surgery, and 20 (15.6%) underwent colostomy. No stent- or surgery-related death occurred in the BTS cohort.

### 3.4. Clinical Outcomes in the PAL Cohort

Treatment was clinically successful in 67 (95.7%) patients in the PAL cohort (n = 70). Similar to the BTS cohort, all enrolled patients in the PAL cohort were subjected to an adverse event assessment (Table 4). Seven (10.0%) early adverse events occurred, including fever in four and stent occlusion in three. Three patients with stent occlusion due to fecal impaction were managed solely by endoscopic procedures. Four patients with fever were followed-up conservatively. Between 20 and 295 days after stent placement, sixteen (22.9%) late adverse events developed, including stent migration in eight, stent occlusion in seven, abdominal pain in one, and fever in one. One patient with stent occlusion underwent surgery 44 days after the stent placement. Additional stents were placed in the remaining six patients with stent occlusion because of growth or overgrowth of the tumor. All eight patients with stent migration were asymptomatic and were followed-up conservatively. One patient with abdominal pain or fever was followed-up conservatively until surgery.

Twenty-three (32.9%) patients, including twenty-two who received chemotherapy and one who received radiotherapy, were treated for primary malignancy after stent placement. Among the patients who received chemotherapy, 15 received molecular-targeted agents such as bevacizumab, panitumumab, and cetuximab. An average of 1.7 (ranging from 1 to 4) chemotherapy regimens were administered during the observation period, and there were 14 cases of partial response (PR) or stable disease (SD).

In the PAL cohort, 29 patients died within the follow-up period, which averaged 219 days (range: 3 to 766 days). Of these, twenty-seven deaths were due to primary disease, one was unexplained, and the remaining one was a stent-related death due to stent occlusion 86 days after stent placement.

## 4. Discussion

In this multicenter prospective safety observational study on colonic stent placement using SEMS with a high axial force zero-border and lower axial force, our findings highlight high clinical and technical success rates (97.0% and 99.5%, respectively), and a remarkably low perforation rate (0.5%), which are pivotal factors in evaluating the safety of this stent in patients with colorectal obstruction. These findings provide a reassuring foundation for clinicians and patients to consider colonic stent placement using SEMS as a therapeutic option.

Safe placement is a major precondition for evaluating the long-term outcomes of colorectal stenting. A high technical success rate can be achieved by improving skills and increasing understanding of the difficulties of colorectal stenting. The procedure is usually performed by endoscopists or surgeons who specialize in colonoscopy and therapeutic pancreaticobiliary endoscopy. Complication rates are reportedly lower when stenting is performed by a physician proficient in endoscopic retrograde cholangiopancreatography [11]. In addition, one study showed that after performing more than 30 colorectal stenting procedures, physicians can repeat the procedure safely and effectively within a brief procedural time in patients with malignant colorectal obstruction [12]. We established the “JCSSPRG”, which is a study group of endoscopists and colorectal surgeons who specialize in colonoscopy and ERCP; regularly held a workshop to discuss colorectal stenting and share tips on stenting; and launched a placement procedure website (http://colon-stent.com/, accessed on 1 August 2021) to increase understanding of the procedure among the members. Consequently, high clinical (92.1–95.5%) and technical (97.8–98.1%) success rates were shown in three prospective studies conducted with our study group [13,14]. Understanding the factors associated with technical difficulty is also important. These include the necessity of multiple stent placements, complete obstruction (CROSS 0), the presence of carcinomatosis, an extra-colonic origin of the tumor, long stricture, and the tumor site in the right colon [15]. In such cases, the stenting procedure should be performed by experts highly experienced in colorectal stenting.

In the 2014 European Society of Gastrointestinal Endoscopy (ESGE) guidelines, SEMS placement was the preferred treatment for PAL, but not as a standard treatment for symptomatic left-sided malignant colorectal obstruction in BTS cases [16]. Based on accumulated evidence, the ESGE guidelines were updated and published in 2020 [1]. It is suggested that stenting as a BTS should be considered within a shared decision-making framework as a treatment option and alternative to emergency surgery for patients with potentially curable left-sided obstructive colon cancer. The ESGE also suggests stenting for malignant proximal colorectal obstruction in either a BTS or PAL setting. Nevertheless, the long-term outcomes of colorectal stenting for malignant colorectal obstructions continue to be a significant clinical concern. With a reported mortality rate as high as 16%, perforation is the most serious complication of colonic stenting [17]. Minor perforations can be treated with fasting and antimicrobial therapy; however, many cases require emergency surgery. Once perforation occurs, the patient’s general condition rapidly deteriorates, often making emergency surgery difficult and resulting in death. Therefore, fatal complications should be avoided. In a systematic review by Watt et al., the perforation rate ranged from 0–83%, but the average was not very high (only approximately 5%) [18]. Interestingly, only one case of perforation (0.5%) was observed in this study, which is a much lower rate than that reported previously. Let us consider the only case of perforation in this study. A patient with malignant colonic obstruction in the splenic flexure and marked dilatation of the small intestine and cecum was treated with a transnasal ileus tube prior to stenting. Stent placement was technically sound and feasible; however, stent expansion was insufficient. Abdominal radiography the next day showed no problems with the stent position; however, full expansion was not achieved, and dilatation of the small intestine and cecum did not improve. Two days after stent placement, computed tomography revealed free air in the cecum, far away from where the stent was placed; therefore, emergency open surgery was performed. Intraoperative findings revealed a tear in the serosa and muscularis propria of the cecum. No perforation was observed at the site of stent placement. The postoperative course was good, and the patient was discharged on the 24th postoperative day, which was not fatal. Perforation may have been avoided if rescue endoscopic interventions, such as additional stent placement or balloon dilatation, had been performed.

In this study, twenty-three (32.9%) patients in the PAL cohort underwent chemotherapy or radiotherapy after stent placement. Furthermore, the present study included fifteen patients treated with molecular-targeted agents such as bevacizumab (seven cases), panitumumab (seven cases), and cetuximab (one case). In addition to the small number of adverse events observed in the present study, the PAL cohort also exhibited no cases of perforation, including in patients given chemotherapy or radiotherapy during the observation period. Some studies have reported that stenting during and after chemotherapy is safe and effective, while others advise against it [19,20]. Additionally, the use of bevacizumab is linked to a higher rate of complications, nearly tripling the risk of perforation [11,21]. With bevacizumab treatment, the perforation rate rises to 15.4%, with a median time to perforation of 21 days [11,21]. Perforation can occur later on during bevacizumab therapy. Considering their functional mechanisms, anti-vascular endothelial growth factor (VEGF) antibody drugs may contribute to perforation. According to the European Society of Gastrointestinal Endoscopy guidelines, antiangiogenic therapy should be considered in patients after colonic stenting; however, during antiangiogenic therapy, colonic stenting should be avoided [1]. Due to the potentially fatal nature of perforation resulting from acute colorectal obstruction, surgical intervention or stenting is necessary, even when patients are undergoing systemic chemotherapy, including bevacizumab therapy. Because the present study did not identify any fatal perforations during chemotherapy or radiotherapy, the SEMSs used in this study may not be associated with chemotherapy-induced perforations. However, further research is needed to clarify the relationship between SEMS placement and chemotherapy, particularly SEMS placement during anti-VEGF antibody drug treatment.

The mechanical properties of SEMSs are another factor that could influence the clinical outcomes of stent placement. Our group has previously evaluated the mechanical properties of several colorectal SEMSs [8]. The JENTLLY Colonic Stent used in this study has an appropriate radial force and a lower axial force, which has contributed to the positive outcomes observed in this study [8]. Furthermore, we introduced a new parameter, “axial force zero-border,” as the angle at which the SEMS’s axial force applied to the intestinal wall becomes almost zero [8]. The SEMS’s axial force increases as the angle of the bent SEMS increases. As the angle of the bent SEMS decreases, the SEMS’s axial force decreases, and it approaches zero at the “axial force zero-border.” Therefore, when the angle of the bent SEMS is greater than the SEMS’s axial force zero-border, the pressure load on the intestinal wall is sustained, which may result in perforation due to intestinal wall injury. The JENTLLY Colonic Stent used in this study exhibited one of the largest axial force zero-border angles, indicating that this SEMS imposes a low sustained load on the intestinal wall, even at large bending angles. Therefore, we assume that this accounts for the lower perforation rate of the SEMS used in this study. Perforations after colonic stent placement generally occur at the edge of the SEMS. Irritation and compression of the colonic mucosa due to axial force from the sharp metal edges of the SEMS are thought to cause perforation. The effect on the colonic mucosa by the SEMS with low axial force and high axial force at the zero-border was considered mild. Consideration of the mechanical properties of SEMSs is crucial to obtain good clinical results.

The design of a SEMS can also impact its technical success rate. Hook-type SEMSs tend to fold in response to longitudinally applied force to the stent’s edge. In contrast, cross-type stents, because they do not fold, might increase the risk of injuries from the stent edge or tip. Recently, the use of hook- and cross-type stents, with lower axial and radial forces, has become widespread in colorectal stenting. The JENTLLY Colonic Stent used in this study is a hook- and cross-type, which has higher release resistance from the delivery system compared to purely cross-structured SEMSs. Consequently, hook- and cross-type SEMS may fail to deploy from the delivery system in cases of tight flexion. In this study, there was indeed one instance where the SEMS could not be deployed.

This study has some limitations. Despite its prospective nature and multicenter collaboration, there may be inherent biases and variations in practice across the participating centers. Additionally, a direct comparison with alternative interventions was beyond the scope of this study. Although this study prospectively assessed a larger patient cohort than in previous studies, it had a single-arm observational design. Future research should include randomized controlled prospective trials that compare the JENTLLY Colonic Stent with other types of stents to confirm its effectiveness and safety. Finally, the administration of chemotherapy after stenting was investigated in a relatively small number of patients. However, the relationship between chemotherapy and perforations in patients undergoing stenting requires further investigation.

In conclusion, our results showed the efficacy and safety of placing SEMSs with low axial force and a high axial force zero-border for malignant colorectal obstruction. The low complication rate with this stent placement, especially the extremely low perforation rate, is remarkable. These findings contribute significantly to the conversation about endoscopic intervention for colorectal obstruction, and stimulate further exploration in this field.

## Figures and Tables

**Table 1:** Baseline patient characteristics (*n* = 199).

Age, years, mean ± SD	73.4 ± 12.5
Sex, *n* (%)	
Male	102 (51.3%)
Female	97 (48.7%)
Height, cm, mean ± SD	158.5 ± 9.8
Body weight, kg, mean ± SD	53.8 ± 11.5
Body mass index, kg/m^2^, mean ± SD	21.3 ± 3.8
ECOG PS, *n* (%)	
0	87 (43.7%)
1	63 (31.7%)
2	35 (17.6%)
3	12 (6.0%)
4	2 (1.0%)
ASA-PS classification, *n* (%)	
1	35 (17.6%)
2	131 (65.8%)
3	32 (16.1%)
4	1 (0.5%)
Symptoms of obstruction, *n* (%)	
Abnormal bowel movements	180 (90.5%)
Bloating	158 (79.4%)
Abdominal pain	135 (67.8%)
Nausea	87 (43.7%)
Vomiting	54 (27.1%)
Etiology of colorectal obstruction, *n* (%)	
Primary colorectal cancer	189 (95.0%)
Metastatic lesion	10 (5.0%)
Treatment history, *n* (%)	
Chemotherapy	3 (1.5%)
Radiotherapy	1 (0.5%)
None	195 (98.0%)
Therapeutic intent, *n* (%)	
Bridge-to-surgery	129 (64.8%)
Palliation	70 (35.2%)
Obstruction/tumor site, *n* (%)	
Right-side colon	53 (26.6%)
Left-side colon	103 (51.8%)
Rectum	43 (21.6%)
Complete/incomplete obstruction, *n* (%)	
Complete obstruction	175 (87.9%)
Incomplete obstruction	24 (12.1%)
Bowel decompression before stent placement, *n* (%)	
None	174 (87.5%)
Nasogastric tube	10 (5.0%)
Transnasal ileus tube	8 (4.0%)
Transanal ileus tube	7 (3.5%)

SD, standard deviation; ECOG, Eastern Cooperative Oncology Group; PS, performance status; ASA-PS, American Society of Anesthesiologists Physical Status.

**Table 2:** Clinical outcomes of stent placement (n = 199).

Technical success, *n* (%)	198 (99.5%)
Technical failure, *n* (%)	
- Failed stent delivery	1 (0.5%)
Procedure time in the technical success cohort, min, mean ± SD	30.0 ± 17.4
Stricture length, cm, mean ± SD	4.3 ± 1.8
Stents placed and strictures, *n* (%)	
Single stricture with 1 stent	189 (95.0%)
Single stricture with 2 stents	9 (4.5%)
Double stricture with 2 stents	1 (0.5%)
Stent diameter, *n* (%)	
First stent	
18 mm	54 (27.1%)
22 mm	145 (72.9%)
Second stent	
18 mm	1 (10.0%)
22 mm	9 (90.0%)
Stent length, *n* (%)	
First stent	
6 cm	30 (15.1%)
8 cm	84 (42.2%)
10 cm	51 (25.6%)
12 cm	34 (17.1%)
Second stent	
6 cm	3 (30.0%)
8 cm	3 (30.0%)
10 cm	4 (40.0%)
Clinical success, *n* (%)	193 (97.0%)
Clinical failure, *n* (%)	
- Technical failure	1 (0.5%)
- Insufficient stent expansion	4 (2.0%)
- Stent occlusion	1 (0.5%)

SD, standard deviation.

**Table 3:** Clinical outcomes in BTS patients (*n* = 128).

Clinical success, *n* (%)	126 (98.4%)
Early adverse events after stent placement, *n* (%)	10 (7.8%)
Stent occlusion	4
Stent migration	1
Perforation	1
Obstructive colitis	1
Fever	2
Abdominal pain	4
Bleeding	0
Intervention for early adverse events	
Emergency surgery	2
Additional stenting	1
Transanal ileus tube insertion	1
No intervention	6
Late adverse events after stent placement, *n* (%)	12 (9.4%)
Stent occlusion	5
Stent migration	3
Perforation	0
Obstructive colitis	1
Fever	3
Abdominal pain	1
Bleeding	0
Intervention for late adverse events	
Transanal ileus tube insertion	1
Transnasal ileus tube insertion	1
No intervention	10
Days from stent placement to surgery, days, mean ± SD	27.6 ± 19.9
Scheduled elective/emergency surgery, *n* (%)	
Scheduled elective surgery	126 (98.4%)
Emergency surgery	2 (1.6%)
Surgical procedures, *n* (%)	
Laparoscopy	103 (80.5%)
Open conversion from laparoscopy	4 (3.1%)
Open surgery	21 (16.4%)
Colectomy, *n* (%)	123 (96.1%)
Colostomy, *n* (%)	20 (15.6%)
CROSS after surgery, *n* (%)	
0	1 (0.8%)
1	1 (0.8%)
2	6 (4.7%)
3	14 (10.9%)
4	106 (82.8%)

BTS, bridge-to-surgery; SD, standard deviation; CROSS, ColoRectal Obstruction Scoring System.

**Table 4:** Clinical outcomes in PAL patients (*n* = 70).

Clinical success, n (%)	67 (95.7%)
Early adverse events after stent placement, *n* (%)	7 (10.0%)
Stent occlusion	3
Stent migration	0
Perforation	0
Obstructive colitis	0
Fever	4
Abdominal pain	0
Bleeding	0
Intervention for early adverse events	
No intervention	7
Late adverse events after stent placement, *n* (%)	16 (22.9%)
Stent occlusion	7
Stent migration	8
Perforation	0
Obstructive colitis	0
Fever	1
Abdominal pain	1
Bleeding	0
Intervention for late adverse events	
Surgery	2
Additional stenting	6
No intervention	8
Treatment for original cancer after stent placement, *n* (%)	
None	47 (67.1%)
Chemotherapy	22 (31.4%)
- Use of molecular-targeted agents	15
Radiotherapy	1 (1.4%)
Duration of follow-up, day, mean ± SD (range)	219 ± 202 (3–766)
Outcomes, *n* (%)	
Alive with no evidence of occlusion	30 (42.9%)
Death due to original cancer	27 (38.6%)
Death due to stent occlusion	1 (1.4%)
Death due to unknown causes	1 (1.4%)
Unknown since last confirmed date	11 (15.7%)

PAL, palliation; SD, standard deviation.

## Data Availability

The original contributions presented in the study are included in the article, further inquiries can be directed to the corresponding authors.
